# Microporous structures on mineralized collagen mediate osteogenesis by modulating the osteo-immune response of macrophages

**DOI:** 10.3389/fbioe.2022.917655

**Published:** 2022-08-29

**Authors:** Jun Li, Xin Luo, Zhao-Yong Lv, Hui-Fen Qiang, Cai-Yao Hou, Kun Liu, Chun-Xiu Meng, Yu-Jue Zhang, Feng-Zhen Liu, Bin Zhang

**Affiliations:** ^1^ Depertment of Oral and Maxillofacial Surgery, School and Hospital of Stomatology, Shandong University & Shandong Provincial Key Laboratory of Oral Tissue Regeneration & Shandong Engineering Laboratory for Dental Materials and Oral Tissue Regeneration, Jinan, Shandong, China; ^2^ Liaocheng People’s Hospital, Liaocheng Hospital Affiliated to Shandong First Medical University, Liaocheng, China; ^3^ Department of Materials Science and Engineering, Liaocheng University, Liaocheng, China

**Keywords:** inflammatory response, mineralized collagen, microporous structures, macrophage polarization, msteogenic differentiation

## Abstract

It is a new hot pot in tissue engineering and regenerative medicine to study the effects of physicochemical properties of implanted biomaterials on regulating macrophage polarization to promote bone regeneration. In this study, we designed and fabricated mineralized collagen (MC) with different microporous structures via *in vitro* biomimetic mineralization method. The microporous structures, mechanical properties, shore hardness and water contact angle measurements were tested. Live/dead cell staining, CCK-8 assay, phalloidine staining, staining of focal adhesions were used to detect cell behavior. ELISA, qRT-PCR, ALP, and alizarin red staining (ARS) were performed to appraise osteogenic differentiation and investigated macrophage response and their subsequent effects on the osteogenic differentiation. The results showed that RAW264.7 and MC3T3-E1 cells were able to survive on the MC. MC with the microporous structure of approximately 84 μm and 70%–80% porosity could promote M2 macrophage polarization and increase the expression level of TGF-β and VEGF. Moreover, the gene expression of the osteogenic markers ALP, COL-1, and OCN increased. Therefore, MC with different microporous structures mediated osteoimmunomodulation in bone regeneration. These data will provide a new idea of biomaterials inducing bone repair and direct the optimal design of novel immune biomaterials, development, and rational usage.

## Introduction

Effective bone tissue repair is critical for all living organisms of the survivals. The physical body is a complex environment, and the implanted biomaterials will inevitably lead to a series of biological reactions. Implantations trigger inflammatory reactions, and the host immune response always leads to failures in clinic (Zhou et al., 2021). Almost all the biomaterials implanted into human beings may induce many host reactions, and the early host reactions and the local microenvironment have an essential influence on bone regeneration and repair (Huang, et al., 2021). After implantation into the body, immune cells acting with the biomaterials surface originate a series of reactions, which decide whether the effective bone repair (Bai et al., 2020; Wei et al., 2022; Gaharwar et al., 2020; Yi et al., 2022). Among all immune cells, macrophages are the primary effector cells because of their significant biodiversity and plasticity. Especially, macrophages are positively involved in the whole stage of bone repair. Depending on context-dependent polarization profiles, macrophage polarized to pro-inflammatory phenotype (M1 macrophage) or pro-tissue regeneration (anti-inflammatory, M2 macrophage) phenotype (Badylak, 2016; Purnell and Hines, 2017; Eming et al., 2017). Therefore, the accurate and quickly conversion from M1 macrophage to M2 macrophage will contribute to positive bone reconstruction and be essential under the bone-forming environment regulating osteoblast differentiation (Brown and Badylak, 2013; Li et al., 2020; Xie et al., 2020).

In the recent, tuning biomaterials properties to modulate macrophage polarization has attracted increasing attention. More and more scholars have turned much attention to how the inflammation response can be controlled to serve the goals of the implantable bracket, particularly with regards to suppressing the immune rejection of exotic bodies and enhancing the integration of scaffolds with native tissue (Chen et al., 2016; Christo et al., 2016; Chen et al., 2017a). Zhao et al. (2020) had demonstrated that calcium-phosphorus phases, with different chemical and physical characteristics modulated the macrophages response (Zhao et al., 2020). Min et al. (2016) found the aggressive inflammatory reaction could be helpful for the origination of osteogenic cascades reaction. However, if the inflammation became overage, it could inhibit the fracture healing (Chen et al., 2018). These indicated that the biomaterials implantations elicited the prominent role of regulating immunoreaction, which should be well guided into them that conduce bone integration. The microporous structures on biological materials were found to have an important moderating role on cell actions. These actions could be operated by adjusting the biophysical performance of the microporous systems (Redlich and Smolen, 2012; Chen et al., 2015). We had previously demonstrated the effects of the surface energy and coarseness of mineralized collagen (MC) implanted on bone absorption. But until now, no document has reported about the microporous structures of MC adjusted macrophage polarization to influence the osteoblast differentiation of MC3T3-E1.

Mineralized collagen (MC) was fabricated bionic mineralization and displayed absolute merit in degradation fast *in vitro*, high hardness, and accelerating osteogenesis differentiation of hMSCs (Xu et al., 2016; Liu et al., 2017; Li et al., 2020; Meng et al., 2021). Shi et al. (2018) had proved that MC was more easily regulated macrophage M2 polarization than HA. Our previous study had demonstrated that the surface roughness of MC regulated the group and single form as well as the production of cell factors, including tumor necrosis factor-α (TNF-α), interleukin-6 (IL-6), interleukin-4 (IL-4) and interleukin-10 (IL-10) from macrophage in a time-dependent manner (Li et al., 2020). As far as we know, whether the microporous structures of MC will influence macrophage polarization and function has not been reported till now.

**FIGURE 1:**
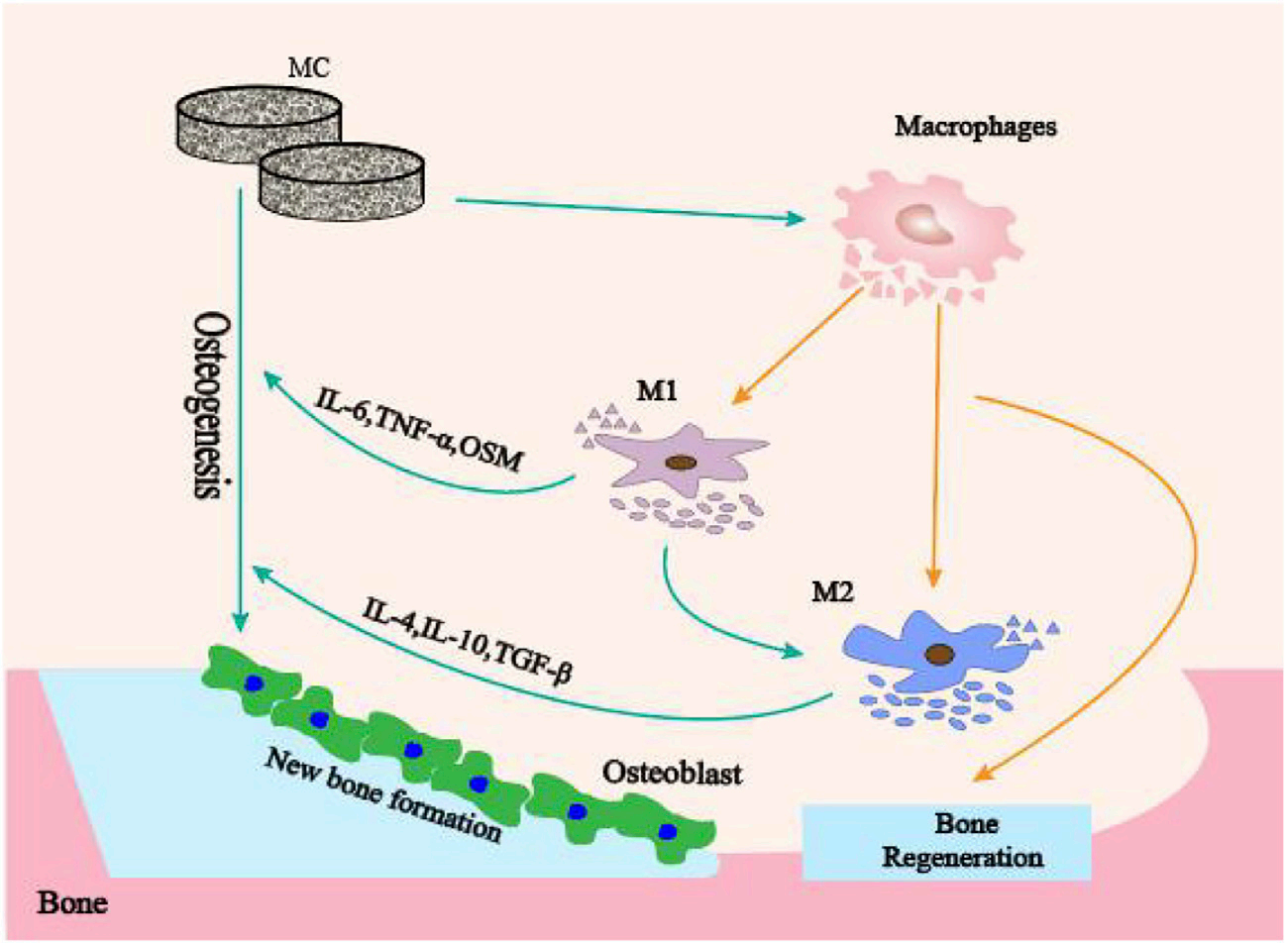
The schematic diagram of MC with different microporous structures regulating macrophage polarization to mediate osteogenesis.

In this study, we investigated the modulatory effects of MC with different microporous structures on bone immune responses to confirm the role of microporous structures on MC mediated bone immune regulation. The schematic diagram of MC with different microporous structures regulating macrophage polarization to mediate osteogenesis was shown in Figure 1. The research will lead to regulating bone immune reaction to induce a reasonable and sufficient osteoimmunology environment for material-mediated bone regeneration, and provide a theoretical basis for developing immunomodulatory biomaterials and bone defect treatment in the clinic.

## Materials and methods

### Preparation of mineralized collagen

Nano-hydroxyapatite (HA)/collagen composites were manufactured by Beijing Allgens Medical Science &Technology Co., Ltd. The products were prepared using purified and deantigenized type I collagen as the template and modulated mineralization on calcium-phosphate solution. The mineral phase was HA containing phosphate, and the crystal size was in the nanometer scale. Type I collagen solution (0.67 g/L) was mixed with a certain proportion of CaCl_2_ and H_3_PO_4_ solutions (Ca/*p* = 1.67). The solution was gently stirred and the pH value was adjusted to 7.4 with sodium hydroxide solution at room temperature. 48 h after the reaction, the precipitate was washed and filtered, and then freeze-dried thoroughly and produced porous mineralized collagen (MC). We prepared MC with large, medium, and small pore sizes, named MC-A, MC-B, and MC-C, with pore sizes of 273 ± 13 μm, 84 ± 3 μm, and 9.7 ± 0.2 μm, and porosities of 80%–90%, 70%–80% and 50%–60%, respectively. The porosity was related to the collagen content. The porosity of the materials were measured by the porosity analyzer. The obtained materials were similar to natural bone in composition and microstructure.

### Characterizations of mineralized collagen

#### Field Emission Scanning Electron Microscopy

The samples were evenly bonded to the conductive adhesive, and a platinum layer was uniformly sprayed by a gold sprayer. The surface morphologies of mineralized collagen (MC) by Field Emission Scanning Electron Microscopy **(**FESEM) (SU-4800, Hitachi, Japan).

### Measurement of compression mechanics

The initial diameter and thickness of each sample were measured by an vernier caliper. Then the piece was placed in the center of the active platform of the electric universal material testing machine (MTS, E44.304, Co., China). Started the oil supply valve of the test machine, the dynamic forum can be quickly lifted. When the sample contacted the upper-pressure plate, the oil supply should be reduced, and the lifting speed should be slowed to avoid the test failure caused by the excessive compression process. Compression speed was 5 mm/min.

### Shore hardness

Took out the shore hardness tester, pointed to zero, and pressed the surface of the sample with the appropriate force and uniform speed. When the end face of the hardness tester was fully contacted with the surface of the model, recorded the value of the hardness tester table. Three independent tests were performed on each material surface.

### Water contact angle measurements

The surface hydrophilicity of MC was analyzed by measuring the contact angle of the material surface. 10 μL of deionized water was added to the surface of the materials. Photographs were taken with a camera within 10 s, and the contact angle was measured by SCA20 software. Three independent tests were carried out on each material surface.

### Cells culture on mineralized collagen with different microporous structures

Mineralized collagen (MC) was cut into discs of 2 mm thickness and then sterilized by irradiation of ^60^Co before use. The RAW 264.7 (mouse monocyte/macnophage) was provided by Liaocheng People’s Hospital. The MC was put at a 24-well plate, and cultured in phosphate buffer saline (PBS, Sangon Biotech) for 4 h before RAW 264.7 seeding. For each sample, the 2×10^5^ RAW 264.7 cells were seeded on the MC. Each substrate was incubated in 1 ml DMEM (Gibco, USA) supplemented with 1% streptomycin/penicillin (Hyclone, USA) and 10% fetal bovine serum (Gibco, USA) for 1, 2 and 3 days, respectively. MC3T3-E1 (mouse embryo osteoblast precursor cells) (ATCC, USA) was inoculated on the surface of different MC in the same way and incubated in α-MEM complete medium for 1, 3 days and osteogenic induction medium (α-MEM complete medium supplemented with 10 nM dexamethasone, 50 μg/ml vitamin C and 10 mM glycerol phosphate) for 7 days, respectively.

### Live/dead cell staining

MC3T3-E1 cells were seeded on MC surface for calcein-acetyl hydroxymethyl ester/propidium iodide (AM/PI) staining at 1 and 3 days, respectively. The living cells (yellow-green fluorescence) and dead cells (red fluorescence) were observed under an inverted fluorescence microscope at 490 ± 10 nm excitation wavelength. The green fluorescence intensity was detected by Image Pro Plus 7.0 for quantitative analysis.

### Cells viability assay

The proliferation of RAW264.7 and MC3T3-E1 were evaluated by a cell counting kit-8 (CCK-8) assay. MC was placed at the bottom of the 48-well plate, and MC3T3-E1 cells were seeded in culture plates at a density of 2×10^4^ cells per well. Cells were incubated in DMEM complete medium for 1 day and replaced with macrophage conditioned medium. After 1, 2, and 3 days of incubation, CCK-8 solution was added to each well for an additional 4 h at 37°C. The cellular activity was assessed by measuring absorbance at a 450 nm wavelength on a microtiter plate reader. In addition, RAW264.7 was seeded on the MC and cultured in DMEM complete medium for 1, 2 and 3 days to detect cellular viability.

### Morphology and micromorphology of macrophagocyte cultured on mineralized collagen

1×10^6^ cells were inoculated in each well, washed with PBS 3 days later, and fixed in 2.5% glutaraldehyde solution for 20 min. Washed three times and then dehydrated with gradient ethanol solution. Finally, placed in a freeze dryer for 1 h and slowly sealed to room temperature. The sample morphology was observed by FESEM.

### Phalloidine staining of F-actin

Cells were fixed with 4% paraformaldehyde for 30 min and permeabilized with 0.1% Triton X-100 for 20 min at room temperature. Samples were blocked with 1% of bovine serum albumin (BSA; Sigma-Aldrich) and incubated with rhodamine labeled phalloidin for 30 min in the dark environment at room temperature, then washed with PBS for 3 times. The samples were also stained with 4′,6-diamidino-2-phenylindole (DAPI) for 30 s in the dark environment at room temperature and washed with PBS 2 times to reveal the nuclei. Cells were observed under a scanning confocal microscope (Nikon, model no. A1R).

### Cell adhesion experiment

#### Staining and imaging of focal adhesions

Cells were fixed (4% paraformaldehyde), permeabilized (0.1% Triton X) and blocked (1% BSA). They were then stained with an anti-vinculin antibody (Abcam, ab129002), followed by staining of a corresponding secondary antibody with Alexa 488. Samples were imaged with a confocal microscope (Nikon, model no. A1R).

### Cytokine determinations

Levels of pro-inflammatory cytokines (TNF-α, IL-1β, IL-6), anti-inflammatory cytokine TNF-β and VEGF were measured with sandwich enzyme-linked immunosorbent assays (ELISAs) as prescribed by the manufacturer (Abcam).

### Quantitative real-time polymerase chain reaction (qRT-PCR)

Cells were collected to extract RNA and reverse transcribed to cDNA using a reverse transcription kit (Shenggong, China). The mRNA expression was quantified using an

ABI 7500 measuring system and SYBR green supermix (Takara, Japan). All data were normalized to GAPDH expression. At the end of the reaction, the 2^−ΔΔCt^ method was used as the relative expression of mRNA according to the last measured Ct value. The primer sequences were shown in Table 1.

**TABLE 1:** Primer sequences used in the study.

Gene	Full name	Primer	Sequences (5′-3′)
iNOS	Inducible nitric oxide synthase	Forward	CAC​CAA​GCT​GAA​CTT​GAG​CG
		Reverse	CGTGGCTTTGGGCTCCTC
Arg-1	Arginine-1	Forward	CTC​CAA​GCC​AAA​GTC​CTT​AGA​G
		Reverse	AGG​AGC​TGT​CAT​TAG​GGA​CAT​C
OCN	Osteocalcin	Forward	CCG​GGA​GCA​GTG​TGA​GCT​TA
		Reverse	AGG​CGG​TCT​TCA​AGC​CAT​ACT
ALP	Alkaline phosphatase	Forward	AGG​GTG​GGT​AGT​CAT​TTG​CAT​AG
		Reverse	GAG​GCA​TAC​GCC​ATC​ACA​TG
OPN	Osteopontin	Forward	ATC​TCA​CCA​TTC​GGA​TGA​GTC​T
		Reverse	TGT​AGG​GAC​GAT​TGG​AGT​GAA​A
COL-1	Collagen-1	Forward	GCTGGAGTTTCCGTGCCT
		Reverse	GACCTCGGGGACCCATTG
GAPDH	Glyceraldehyde-3-phosphate dehydrogenase	Forward	TGA​CCA​CAG​TCC​ATG​CCA​TC
		Reverse	GAC​GGA​CAC​ATT​GGG​GGT​AG

### Preparation of macrophage conditioned medium and effect on MC3T3-E1 osteogenesis

MC with different microporous structures were spread in 6-well plates. For each sample, 1×10^6^ cells were seededon the materials surface. Macrophages and materials were co-cultured in complete DMEM medium for 3 days. The supernatant was collected and centrifuged by 1,000 rpm for 5 min to obtain conditioned medium (CM). MC3T3-E1 cells were plated at a density of 2×10^3^ cm^−2^. DMEM complete medium was replaced by CM osteogenic induction medium after 1 day. Then the effect of CM on MC3T3-E1 osteogenesis was detected by qRT-PCR, alkaline phosphatase (ALP) staining and activity analysis, alizarin red S staining, and activity analysis.

### ALP staining and activity analysis

After 7 days of MC3T3-E1 culture, ALP expression was detected by 5-Bromo-4-Chloro-3-Indolyl Phosphate/Nitrotetrazolium Blue chloride (BCIP/NBT) Alkaline Phosphatase Color Development Kit (Beyotime, C3206). The color development solution was added sequentially according to the kit instructions, incubated at room temperature without light for 15 min, and then observed and taken photos under the microscope. ALP activity was measured according to the teachings of the Alkaline phosphatase assay kit (Nanjing Jiancheng bioengineering institute).

### Alizarin red S staining and activity analysis

MC3T3-E1 was fixed with 4% paraformaldehyde for 10 min after 21 days in CM osteogenic induction medium and incubated with 0.2% alizarin red S staining solution for 30 min at room temperature. After drying, 10% dodecyl pyridine chloride was added and incubated at room temperature for 1 h. The absorbance at 562 nm was measured on a 96-well plate.

### Statistical analysis

All the analyses were performed using the software SPSS 22.0 (IBM SPSS, Armonk, New York, United States). All the data were expressed as means ± standard deviation (SD). Statistical analysis was determined by one-way analysis of variance (ANOVA) and Tukey’s post-hoc test. A level of significance was set at *p* < 0.05.

## Results

### Characterizations of different mineralized collagen

**FIGURE 2:**
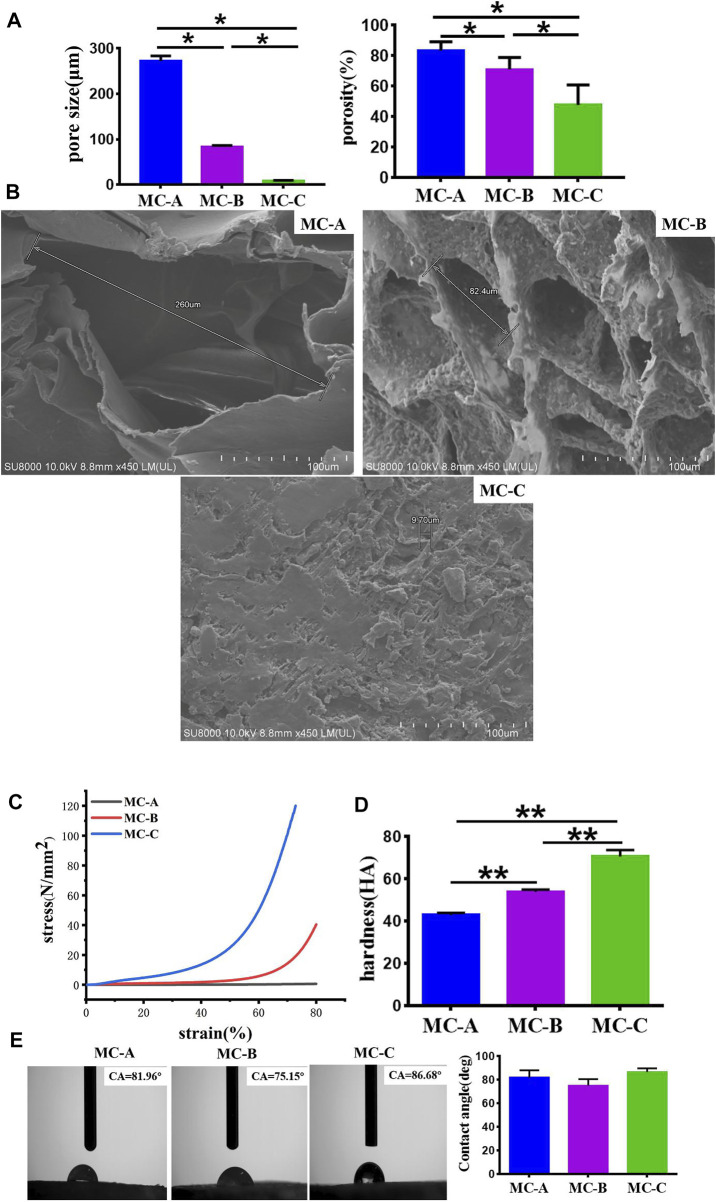
Characterizations of mineralized collagen. **(A)** The pore size and porosity of MC-A, MC-B, and MC-C; **(B)** FESEM micrographs of MC-A, MC-B, and MC-C (scale bar:100 μm); **(C)** Compression mechanics of MC-A, MC-B, and MC-C**; (D)** Shore hardness of MC-A, MC-B and MC-C**; (E)** Water contact angle of MC-A, MC-B, and MC-C**.** Data are presented as mean ± SD. *p < 0.05; **p < 0.01*.*

We had characterized the three groups of mineralized collagen (MC) with microporous structures, the results were as shown in Figure 2. From Figure 2Afig2, the results showed that the pore size of MC-A was 273 ± 13 μm and porosity was 80–90%, MC-B was 84 ± 3 μm, and porosity was 70%–80%, and MC-C was 9.7 ± 0.2 μm, and porosity was 50%–60%. From FESEM micrographs (Figure 2B), many pores were distributed in the scaffolds with good interconnectivity. According to our previous cooperative research, the porosity of MC was about 70%, and the aperture size was 50–300 μm, which were incredibly similar to the natural bone in both composition and microstructure, and had excellent biocompatibility and biodegradable (Xu et al., 2016; Li et al., 2020). The hydrophilicity, shore hardness, and compression mechanics of MC scaffolds with different pore sizes were tested. The results showed no significant difference in hydrophilicity among the three groups. The compressive strength and shore hardness of MC increased with the decrease of pore sizes (Figures 2C–E).

### Effects of MC with different microporous structure on proliferation, morphology, and osteogenic differentiation of MC3T3-E1

**FIGURE 3:**
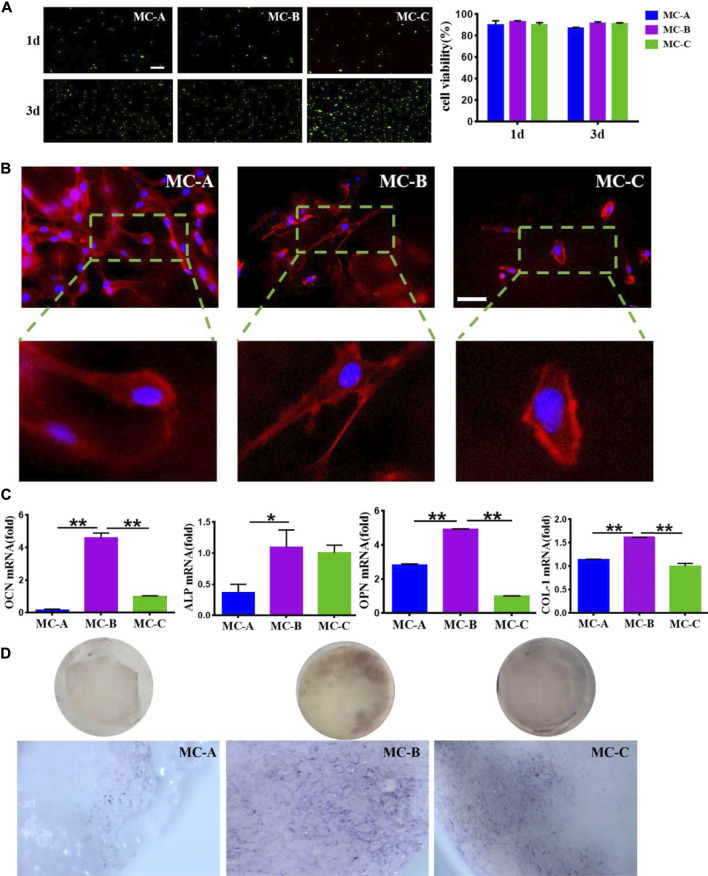
Effects of MC-A, MC-B, and MC-C on MC3T3-E1 cells. **(A)** Live/dead cell staining (scale bar: 100 μm); **(B)** Effects of MC-A, MC-B, and MC-C on the morphology of MC3T3-E1 cells were detected by rhodamine labeled phalloidin staining (scale bar: 50 μm); **(C)** The expression of the osteogenic gene was detected by qRT-PCR; **(D)** ALP staining (stereomicroscope, x60). Data are presented as mean ± SD. *p < 0.05; **p < 0.01*.*

We explored the effects of MC with different microporous structures on the activity, morphology, and osteogenic differentiation of MC3T3-E1. The typical field of vision an inverted fluorescence microscope was selected to observe the staining of live and dead MC3T3-E1 cells at 1 and 3 days. After 1 day, live green cells were predominant in the field of view. On day 3, the number of live cells on the surface of MC increased in all three groups compared with day 1, and the number was significantly more than that of dead cells (Figure 3A). Cells inoculated in MC-A and MC-B with interconnected pseudopods and clear microfilaments. Cells inoculated in MC-B had an increased extension area and enhanced extension ability. Cells inoculated in MC-C had an irregular shapes and bundle-like microfilaments (Figure 3B). OCN is a specific non-collagen protein in bone matrix, which can understand the activity of osteoblasts by OCN level (Komori, 2020). ALP is one of the phenotypic markers of osteoblasts, which can directly reflect the activity or functional status of osteoblasts (Annibali et al., 2021). OPN is involved in bone matrix mineralization and resorption processes (Akram et al., 2018). COL-1 is the most predominantly expressed product during the proliferative phase of osteoblasts (Lin et al., 2019). Therefore, MC3T3-E1 cells were inoculated on MC and cultured under osteogenic induction conditions at 7 days. The expression of osteogenic related genes (ALP, COL-1, OCN, and OPN) was detected by qRT-RCP. It was found that the gene expression level was notably increased in the MC-B group (Figure 3C). Moreover, the MC-B group showed intense ALP staining (Figure 3D). These results suggested that the MC-B group promoted osteogenic differentiation of MC3T3-E1.

### Effects of mineralized collagen with different microporous structure on macrophage morphology

**FIGURE 4:**
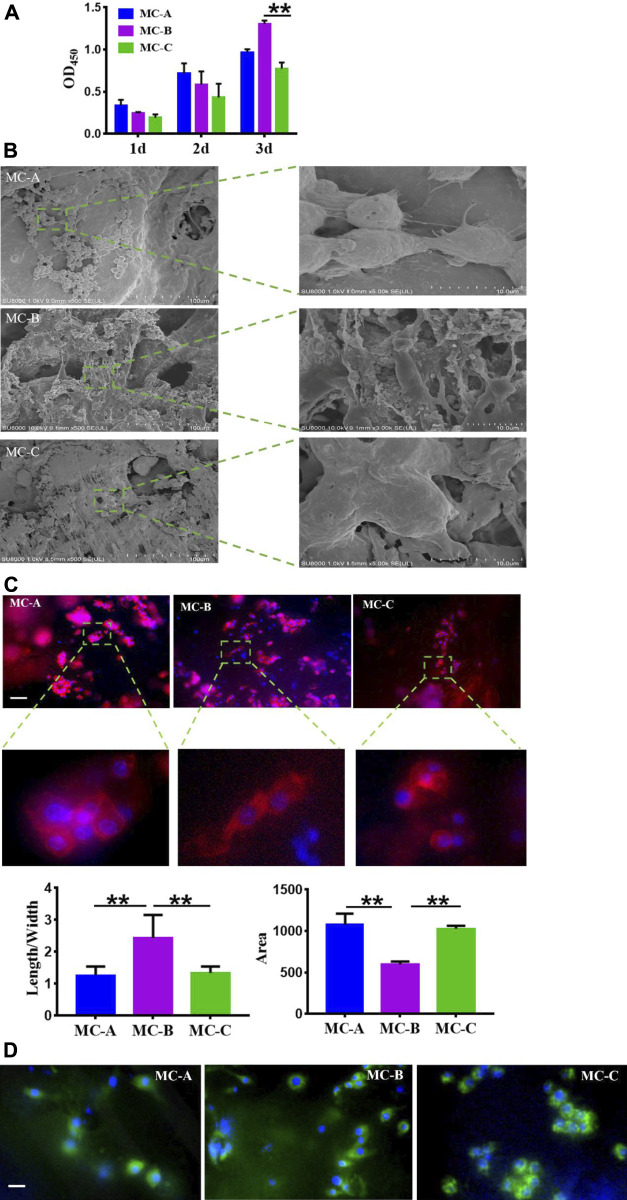
Effects of mineralized collagen with different microporous structures on macrophages morphology. **(A)** CCK-8 cell proliferation experimentation of RAW 264.7 cells after cultured 1 d, 2 d, and 3 days *in vitro*; **(B)** Population and individual morphology of macrophages grown on different mineralized collagen (scale bar:100 μm); **(C)** Detection of the cytoskeleton morphology of macrophages cultured for 3 days by rhodamine labeled phalloidin staining (scale bar: 50 μm); **(D)** Immunofluorescence detection of focal adhesion morphology of macrophages after 3 days of co-culture (scale bar: 50 μm).

To explore the possible role of macrophages in mineralized collagen (MC)-mediated bone formation, RAW264.7 cells were seeded on MC with different pore sizes to analyze the changes of macrophages. The proliferation of RAW264.7 cells inoculated on MC for 1, 2, and 3 days was shown in Figure 4A. We found that the OD values of each group increased gradually with the increase of culture time. Still the cell proliferation ability of the MC-B group was significantly higher than that of the MC-C group at 3 days. The morphology of macrophages in three groups was shown in Figure 4B after 3 days of culture on each surface. Focal adhesion are the sites where cytoskeleton and signal protein structure adhere to extracellular matrix. These signal proteins include vinculin, integrin family members and tyrosine kinase family members. Among them, due to the rich content of vinculin, the change of adhesion point can be accurately reflected by measuring the expression of vinculin (Han, et al., 2021). From Figures 4C,D, we can see the cytoskeleton morphology and the focal adhesions morphology of macrophages cultured in three groups at 3 days. Based on the above results, there was found that the macrophages in the three groups displayed different shapes. The three groups of RAW264.7 cells all stretched out pseudopods to connect with the biomaterials. On the surface of MC-A, the adherent cells had a sizeable spreading area and different adherent cell morphology, some of which were spindle-shaped or round. On the surface of MC-B, the spreading area of adherent cells was small, and the cells were shuttle-shaped, which was a typical morphology of M2 cells (Liu et al., 2022), on the MC-C surface, the morphology of adhesion cells was irregular and without migrating into the collagen network. RAW264.7 survived on the surface of all three groups of materials, but the medium pore size MC-B promoted cell polarization to M2 macrophage.

### Effects of mineralized collagen with different microporous structure on macrophage polarization

**FIGURE 5:**
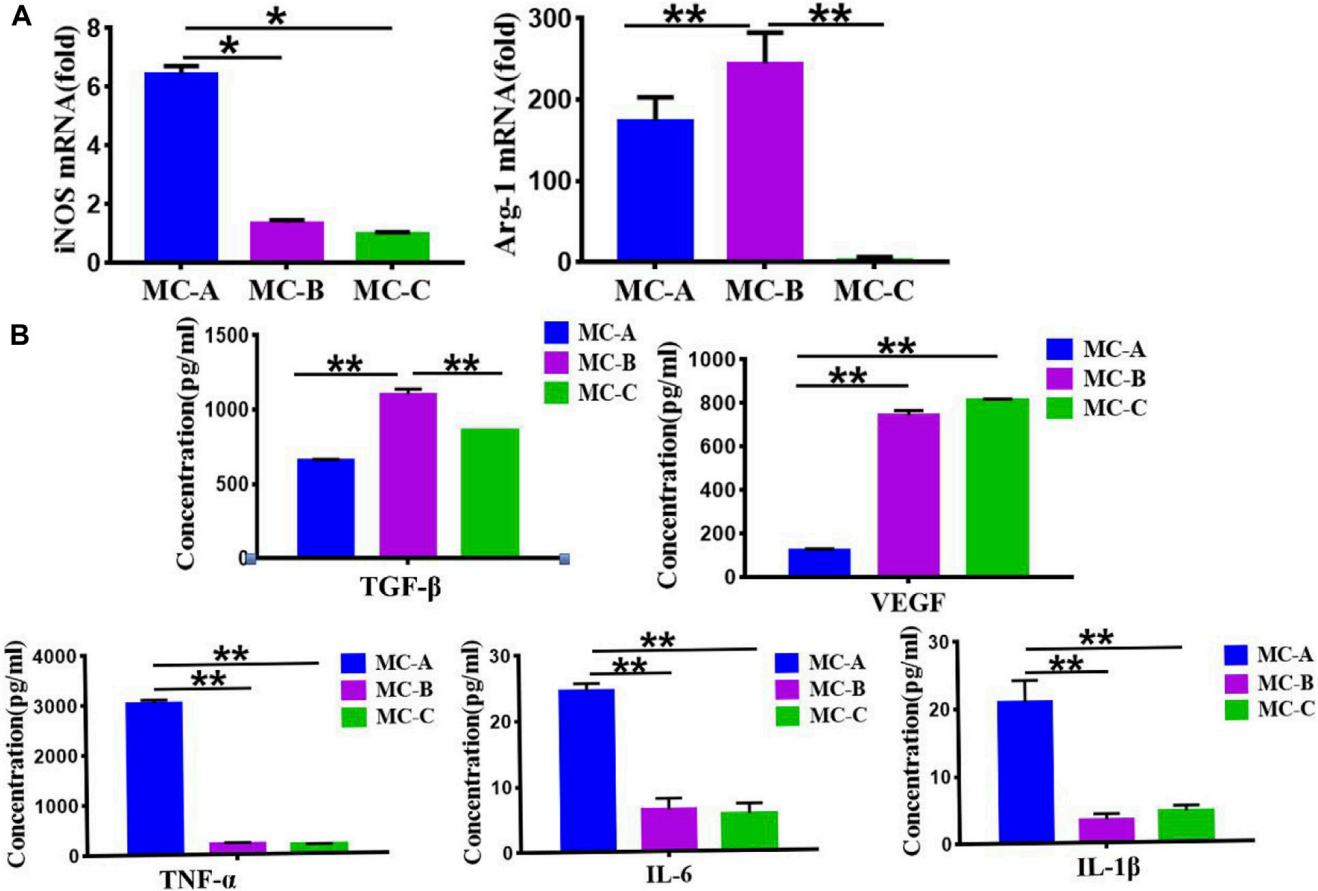
Effects of mineralized collagen with different microporous structures on macrophages polarization. **(A)** Expression of iNOS and Arg-1 marker showing the M1 and M2 polarization of macrophages cultured (M1: iNOS; M2: Arg-1); **(B)** Secretion of TGF-β, VEGF, and proinflammatory cytokines (TNF-α, IL-6, IL-1β). Data are presented as mean ± SD. *p < 0.05; **p < 0.01.

Figure 5A showed that the MC-B group increased the expression of Arg-1 (a characteristic marker of M2 macrophage) while the expression of iNOS (a characteristic marker of M1 macrophage) (Li et al., 2019) was significantly increased in the MC-A group. Figure 5B showed that the expression of anti-inflammatory cytokine TGF-β and growth factor VEGF were significantly higher in the MC-B group. In comparison the expression of pro-inflammatory cytokines (TNF-α, IL-1β, IL-6) was considerably lower in MC-B and MC-C than in MC-A. According to the above results, we inferred that MC-B promoted MC3T3-E1 osteogenic differentiation by promoting macrophage M2 polarization.

Osteogenic Differentiation of MC3T3-E1 Cells on Mineralized Collagen with Different Microporous Structures under Osteoimmune Environments

**FIGURE 6:**
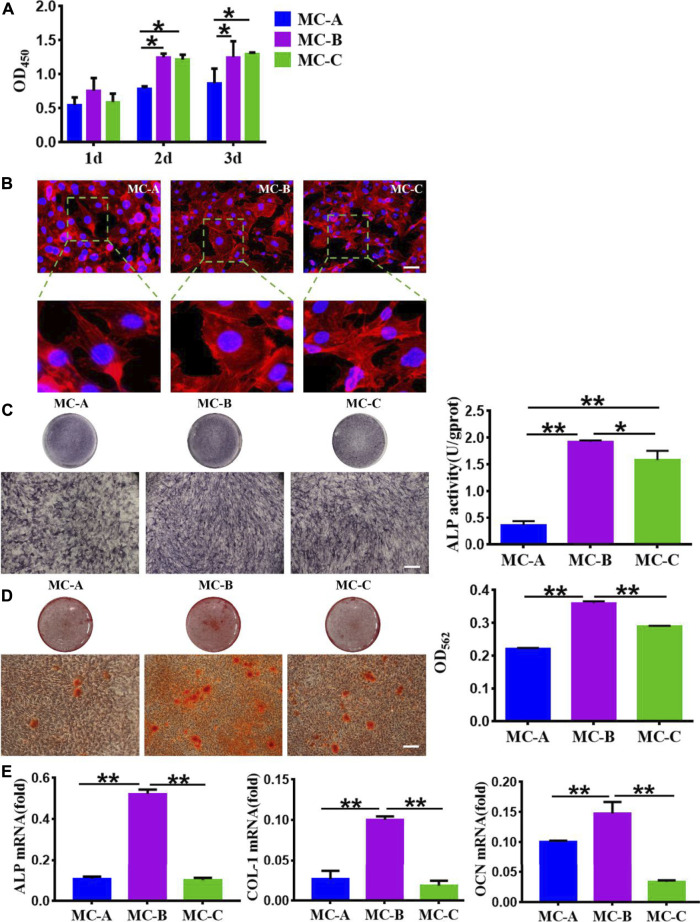
Effect of macrophage conditioned medium produced by mineralized collagen with different pore sizes on MC3T3-E1. **(A)** CCK-8 cell proliferation experimentation of MC3T3-E1 cells after cultured 1d, 3d, and 7 days *in vitro*; **(B)** The cytoskeleton morphology of MC3T3-E1 cells cultured on different groups (scale bar: 50 μm); **(C)** ALP staining and quantification in MC3T3-E1 cells (scale bars:100 μm); **(D)** Alizarin red S staining and quantification in MC3T3-E1 cells (scale bars:100 μm); **(E)** The expression of osteogenic gene was detected by qRT-PCR. Data are presented as mean ± SD. *p < 0.05;**p < 0.01.

Based on the osteo-immune responses of macrophages, we investigated the effect of macrophages on osteogenic differentiation of MC3T3-E1 cells. Three conditioned media for co-culture of mineralized collagen and macrophages were taken to incubate MC3T3-E1 cells, the results showed in Figure 6. From Figure 6A, we can see that the proliferation of MC3T3-E1 cells incubated in the MC-B, MC-C conditioned medium with small pore size was significantly higher than that of MC-A with a large pore size at 2 and 3 days. Phalloidin staining showed that MC3T3-E1 cells in the MC-B group were densely distributed with large spreading area and clear microfilaments compared with the MC-A and MC-C groups (Figure 6B). We assessed the osteogenic ability of the three groups of MC3T3-E1 cells by ALP staining, alizarin red staining, and osteogenic-related gene expression. The results showed that the MC-B group had more ALP positive cells (Figure 6C), more calcium salt deposition (Figure 6D), and higher expression levels of osteogenic-related genes (Figure 6E). Therefore, we inferred that MC-B could induce macrophage polarization toward the M2 type, which had better performance than MC-A and MC-C in stimulating the inflammatory response of the organism and was more conducive to bone reconstruction. In response to MC-B, M2 macrophage generated osteoimmunology microenvironment, which resulted in outcomes that guide bone regeneration in some situations.

## Discussion

The osteoimmune environment plays an essential role in bone repair, most of the repair process promotes the differentiation of osteoblastic cells on the implanted-biomaterial surfaces generated the microenvironment, but the topics about microporous structures-mediated osteogenesis are generally neglected (Liu, et al., 2010; Sussman, et al., 2014; Niu, et al., 2020; Sun, et al., 2022). The presence of microporous structures on the biomaterial surface is crucial for bone formation (Kang, et al., 2021). In our study, we investigated the macrophage responses of microporous structures on mineralized collagen (MC) with different porosity and sized pores and the subsequent effects on the osteogenic differentiation of MC3T3-E1 cells. We first analyzed the effect of MC with different microporous structures on MC3T3-E1 cells osteogenic differentiation. It was shown that MC with medium pore size (MC-B) enhanced the extension area and extension capacity as well as osteogenic differentiation of MC3T3-E1 cells. The polarization of macrophage is very sensitive to the physical and chemical properties of biomaterials. These M1 macrophage and M2 macrophage can mediate the host immune response to implanted scaffolds and exert the potential to different degrees in bone regeneration and repair (Chen et al., 2017b; Duan, et al., 2019). We analyzed the effect of microporous structures on macrophage behavior. When RAW 264.7 macrophages were cultured on MC, macrophages maintained surface marker expression and polarized to M1 and M2 phenotypes. Our results indicated that on the surface of MC-B, macrophages polarized into spindle-shaped M2 phenotype. The enhanced M2 polarization on MC-B was further confirmed by qRT-PCR. The expression of M2 type surface marker Arg-1 was the most obvious in RAW264.7 co-cultured with MC-B. In MC with a large pore size (MC-A), the expression of both was reversed. The results of ELISA showed that typical pro-inflammation factors, e.g., IL-6, TNF-α, and IL-1β, were up-regulated on MC-A while growth factors and anti-inflammatory factors, e.g., VEGF and TGF-β, were up-regulated on MC-B. ALP staining, alizarin red staining, and qRT-PCR revealed that MC-B significantly enhanced M2 macrophage polarization and subsequently M2 macrophage mediated osteogenic differentiation of MC3T3-E1 cells.

In this study, it was found that the microporous structures and the pore size of biomaterial implantations may be important adhesive cues, affect the spreading and cell shape of macrophages, and modulate the inflammatory response. Because immune cells are frontline cells attingent with inserted biomaterials, their reaction and the immune microenvironment they generate are essential to determine biomaterial-mediated osteogenesis. These impacts demonstrate that biomaterial mediated immunoreaction performed a critical role in micropore structure-mediated osteogenesis (Liao, et al., 2021).

It is essential for the crosstalk between immune cells and the bone forming cells to complete the inflammation stage and initiate the new bone formation (Liu et al., 2022; Yu et al., 2022). Our results indicated the osteoimmunomodulatory property of the microporous structures MC with different sized pores and its critical effects on bone regeneration. This study suggested that osteogenic differentiation of bone cells was not only determined by the nature of biomaterial implantations, but also influenced by the inflammatory environment generated by the interaction of immune cells and biomaterial implantations significantly. Therefore, creating an osteoimmunology environment that stimulate osteogenesis by biomaterials with optimal design and development and rational application is vital in bone tissue and regeneration.

The early immune environment decides the follow-up action of bone cells (Fernandes et al., 2017; Chen et al., 2018; Sadowska et al., 2018). Nonetheless, better comprehending of this relation requires further *in vitro* investigation of signalling pathways responsible for enhanced osteogenic differentiation of bone forming cells (Fan et al., 2021). Therefore, further *in vivo* experiments designed to research the interaction between immune and bone cells should be conducted to sufficient support driven presupposition.

## Conclusion

Microporous structures have apparent regulatory effects on macrophages responses. The osteo-immune environment promoted by the mineralized collagen (MC) with about 85 μm microporous structure and 70% porosity was conducive to the osteogenic differentiation of MC3T3-E1 cells, suggesting a favorable osteo-immunomodulatory effect, which could be favorable for increasing the osteogenesis capability of biomaterials for bone regeneration. Microporous structures on MC elicited notable influence on regulating the immunological reaction. The induced osteoimmunology environment significantly regulated osteoblast differentiation, which may suggest a new orientation for systemic research. We need further study to acquire more particulars on the biomaterials dependent response of macrophages on the level of the molecular and the function of immunoregulatory function of biomaterials in bone tissue and engineering. The science gained from this research can supply clues for the intending development of improved immune therapy for bone biomaterials applications and highlight emerging concepts that may expand therapeutic perspectives in bone repair and regeneration.

## Data Availability

The original contributions presented in the study are included in the article/Supplementary Material, further inquiries can be directed to the corresponding authors.
